# Supplementary material for: A hypothalamic circuit for anticipating future changes in energy balance
Source: bioRxiv. 2025 Sep 27:2025.09.27.678865. Preprint. [Version 1] doi: 10.1101/2025.09.27.678865 (PMC12485711; doi:10.1101/2025.09.27.678865)
Supplement: 1 [file NIHPP2025.09.27.678865V1-supplement-1.pdf]

bioRxiv preprint doi: https://doi.org/10.1101/2025.09.27.678865; this version posted September 28, 2025. The copyright holder for this preprint (which was not certified by peer review) is the author/funder, who has granted bioRxiv a license to display the preprint in perpetuity. It is made available under aCC-BY 4.0 International license.

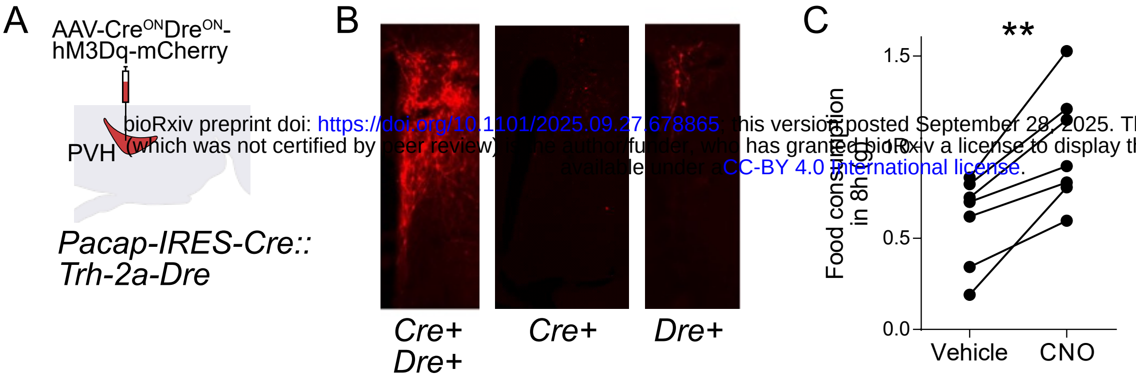

Figure S1

bioRxiv preprint doi: https://doi.org/10.1101/2025.09.27.678865; this version posted September 28, 2025. The copyright holder for this preprint (which was not certified by peer review) is the author/funder, who has granted bioRxiv a license to display the preprint in perpetuity. It is made available under aCC-BY 4.0 International license.

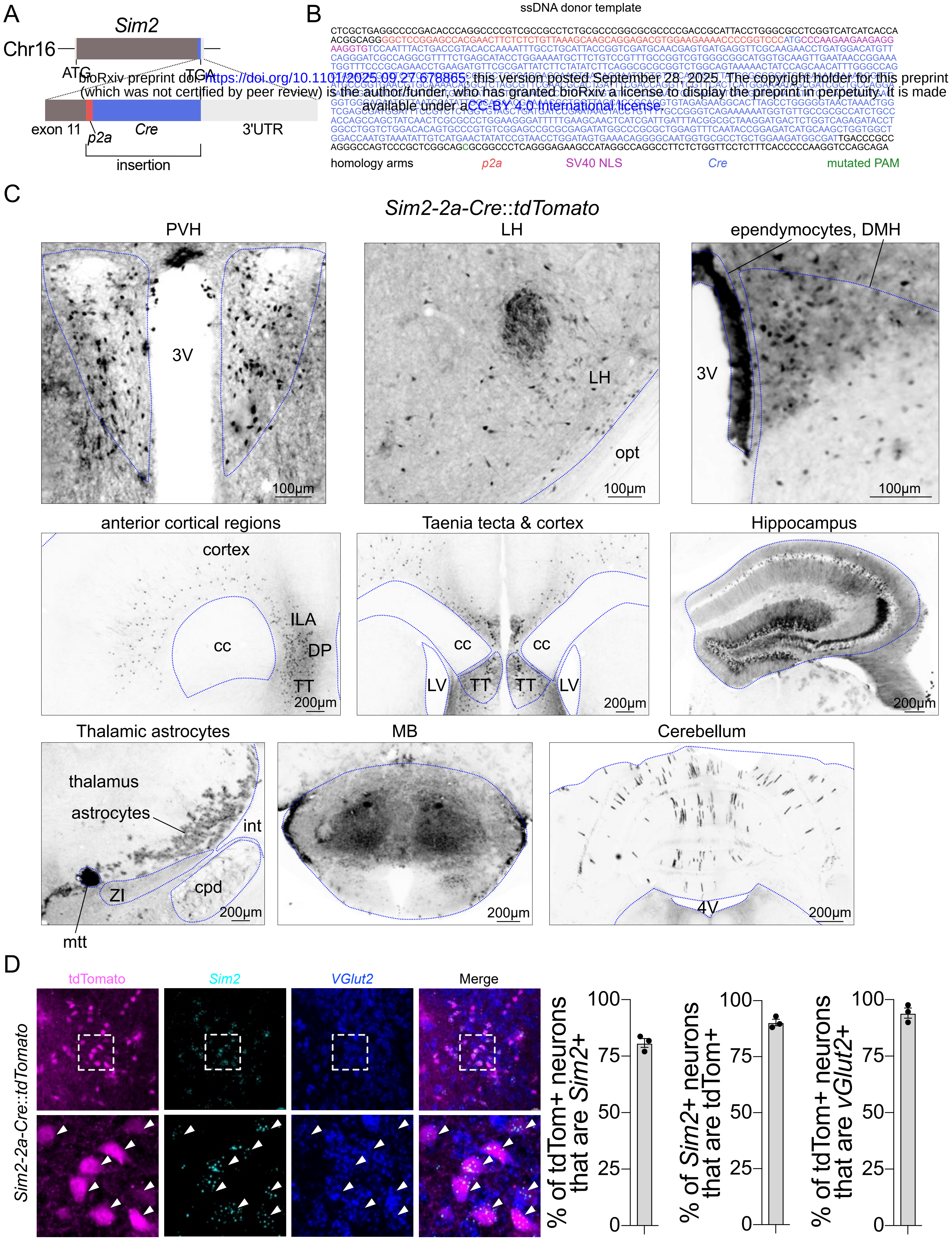

Figure S2

bioRxiv preprint doi: https://doi.org/10.1101/2025.09.27.678865; this version posted September 28, 2025. The copyright holder for this preprint (which was not certified by peer review) is the author/funder, who has granted bioRxiv a license to display the preprint in perpetuity. It is made available under aCC-BY 4.0 International license.

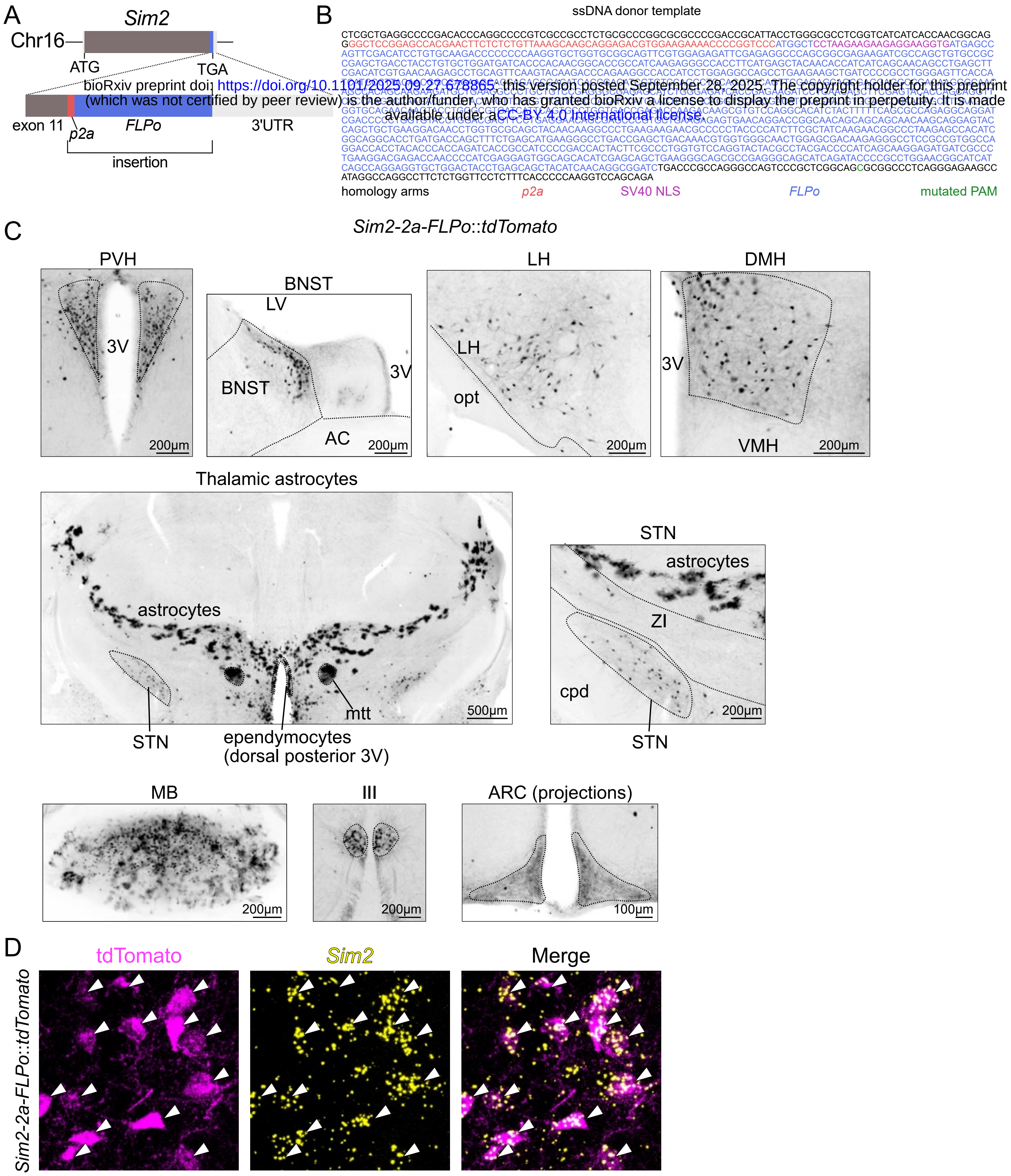

Figure S3

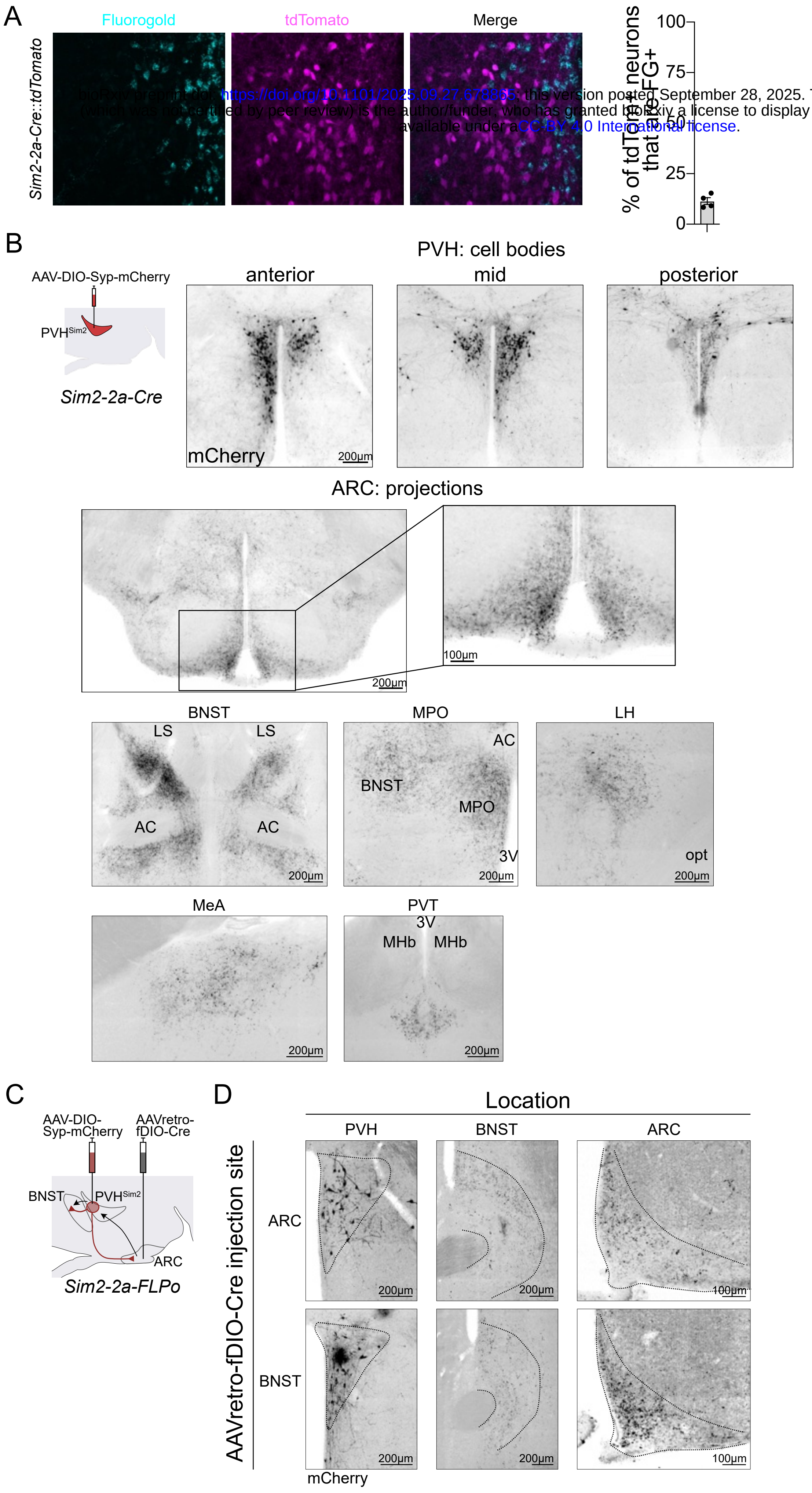

Figure S4

bioRxiv preprint doi: https://doi.org/10.1101/2025.09.27.678865; this version posted September 28, 2025. The copyright holder for this preprint (which was not certified by peer review) is the author/funder, who has granted bioRxiv a license to display the preprint in perpetuity. It is made available under aCC-BY 4.0 International license.

bioRxiv preprint doi: https://doi.org/10.1101/2025.09.27.678865; this version posted September 28, 2025. The copyright holder for this preprint (which was not certified by peer review) is the author/funder, who has granted bioRxiv a license to display the preprint in perpetuity. It is made available under aCC-BY 4.0 International license.

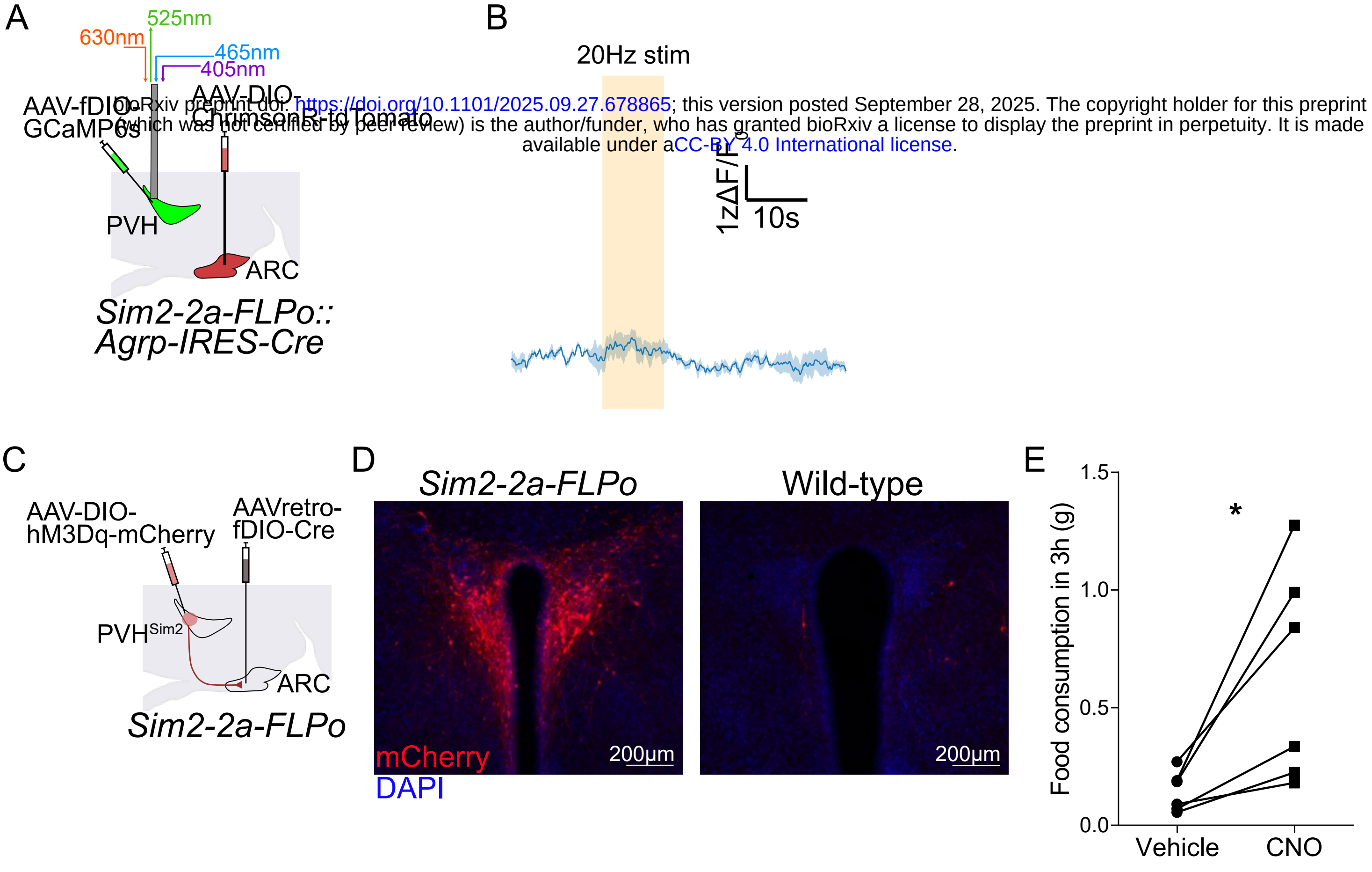

Figure S5

bioRxiv preprint doi: https://doi.org/10.1101/2025.09.27.678865; this version posted September 28, 2025. The copyright holder for this preprint (which was not certified by peer review) is the author/funder, who has granted bioRxiv a license to display the preprint in perpetuity. It is made available under aCC-BY 4.0 International license.

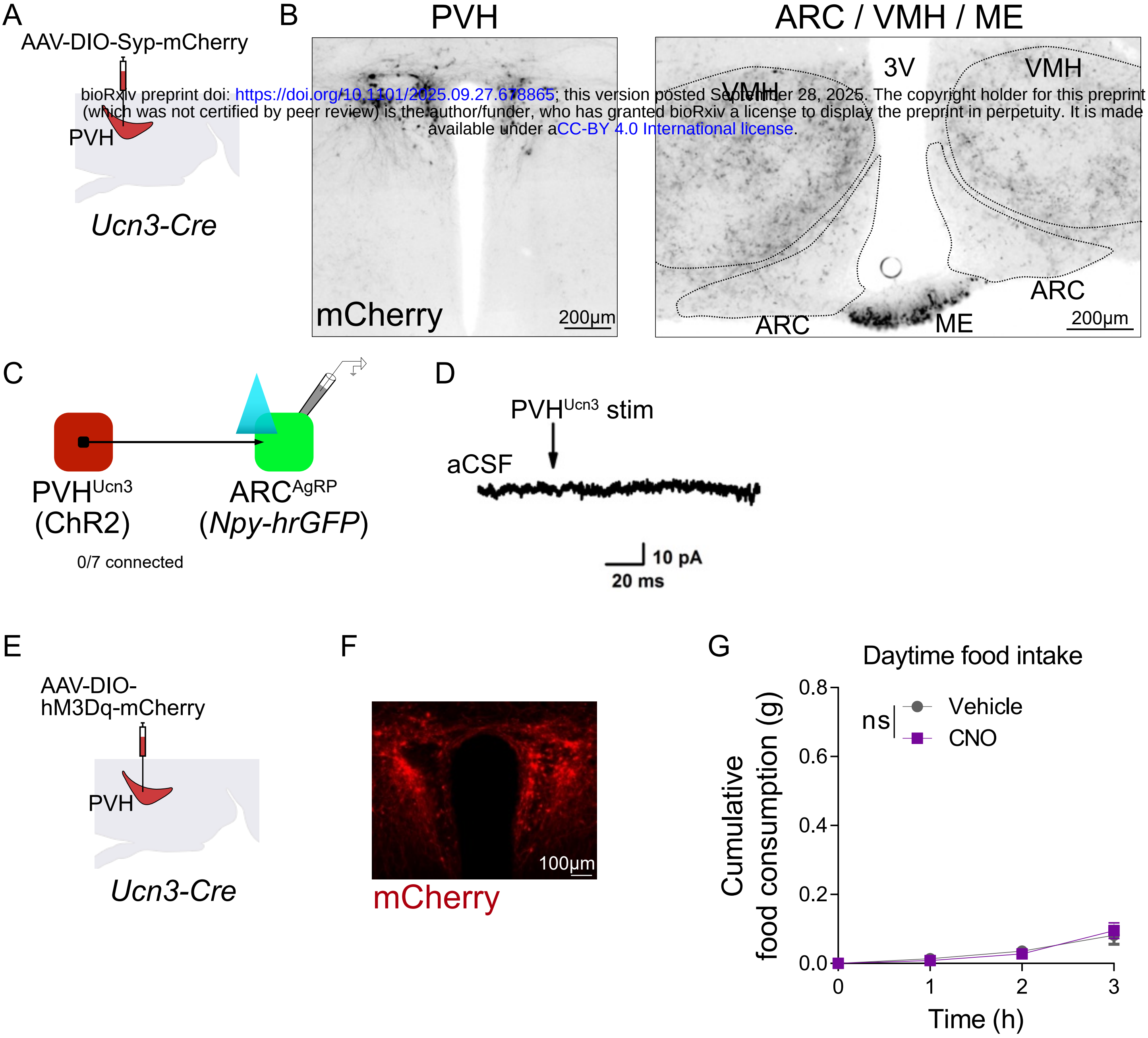

Figure S6

bioRxiv preprint doi: https://doi.org/10.1101/2025.09.27.678865; this version posted September 28, 2025. The copyright holder for this preprint (which was not certified by peer review) is the author/funder, who has granted bioRxiv a license to display the preprint in perpetuity. It is made available under aCC-BY 4.0 International license.

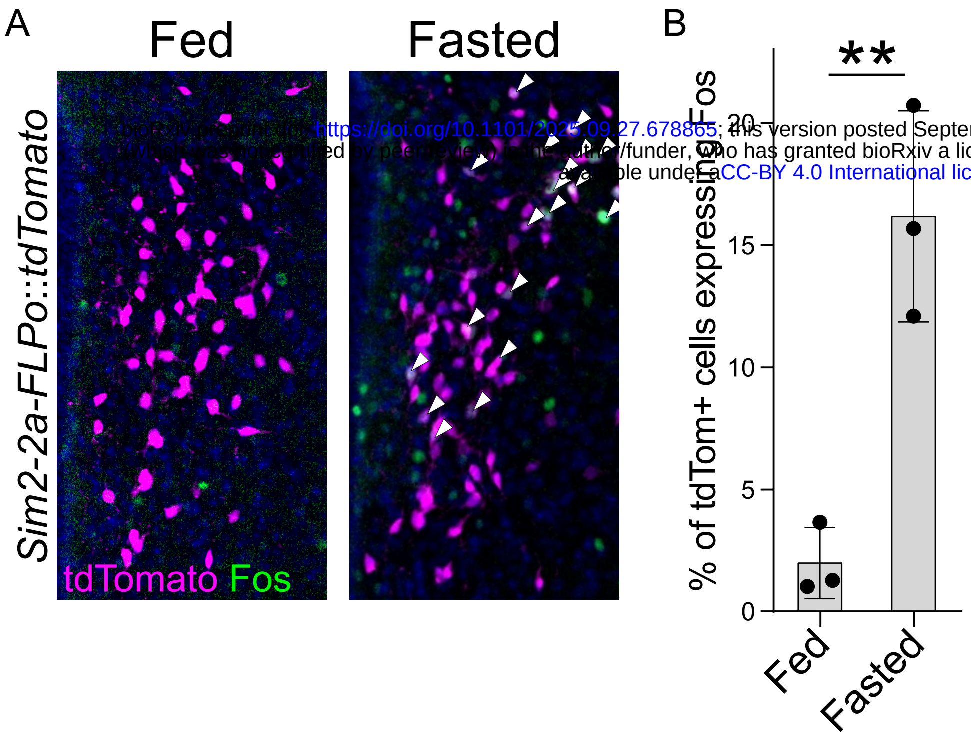

Figure S7

bioRxiv preprint doi: https://doi.org/10.1101/2025.09.27.678865; this version posted September 28, 2025. The copyright holder for this preprint (which was not certified by peer review) is the author/funder, who has granted bioRxiv a license to display the preprint in perpetuity. It is made available under aCC-BY 4.0 International license.

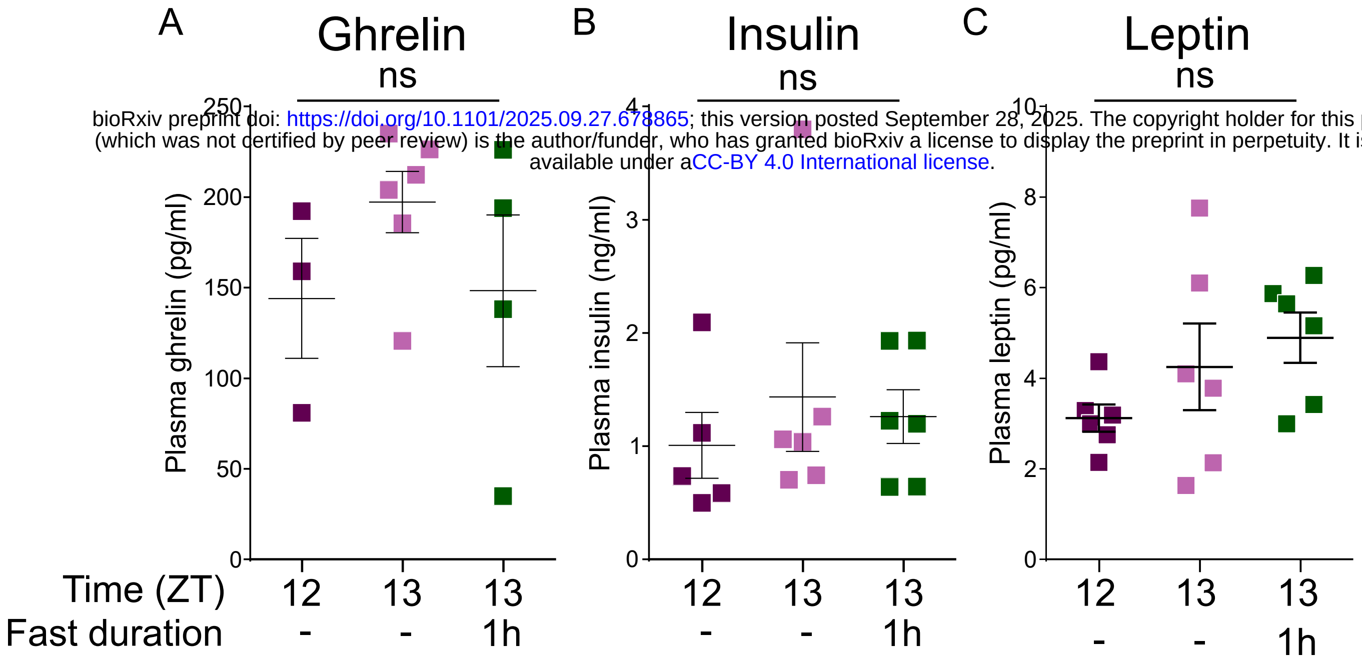

Figure S8

bioRxiv preprint doi: https://doi.org/10.1101/2025.09.27.678865; this version posted September 28, 2025. The copyright holder for this preprint (which was not certified by peer review) is the author/funder, who has granted bioRxiv a license to display the preprint in perpetuity. It is made available under aCC-BY 4.0 International license.

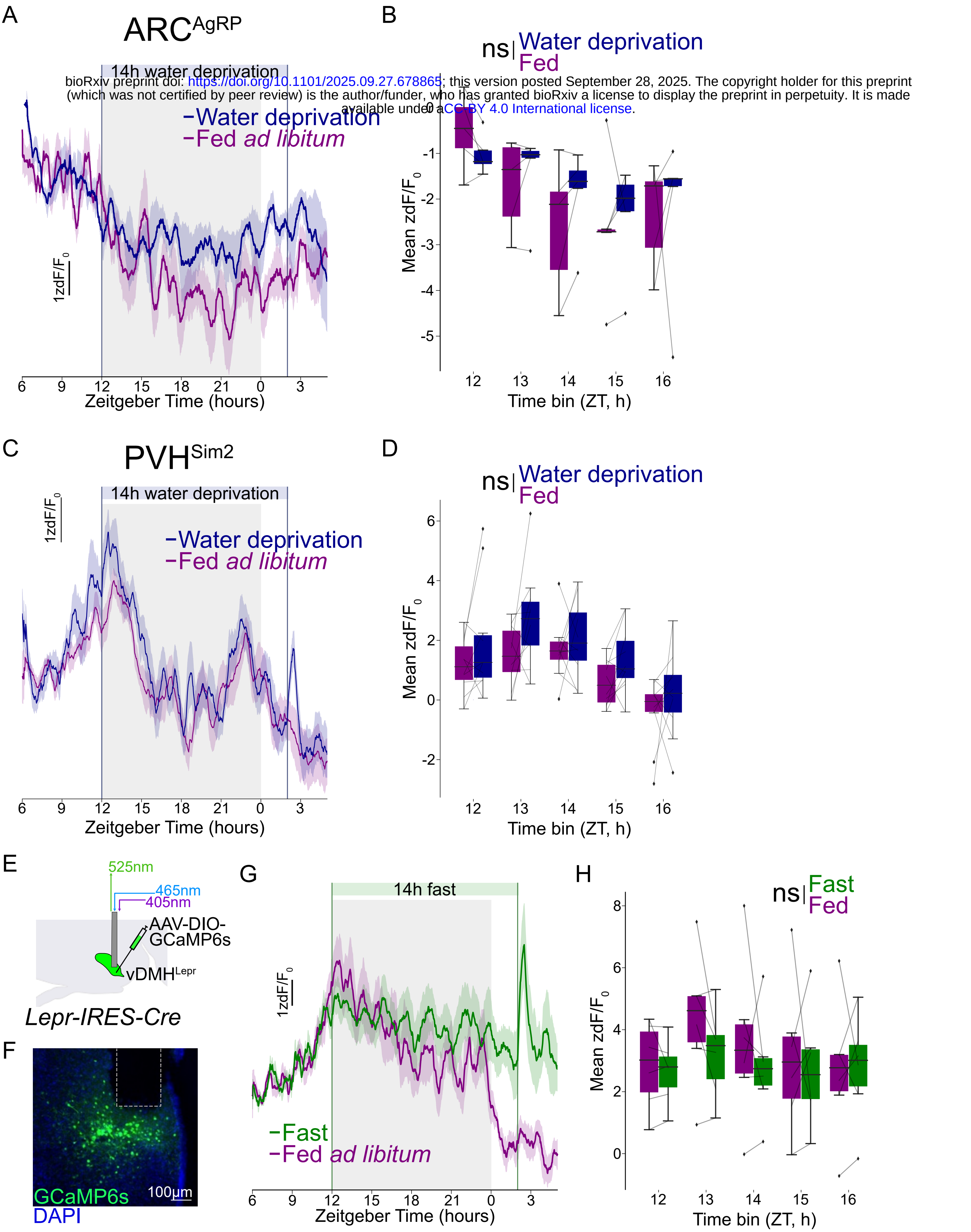

Figure S9

bioRxiv preprint doi: https://doi.org/10.1101/2025.09.27.678865; this version posted September 28, 2025. The copyright holder for this preprint (which was not certified by peer review) is the author/funder, who has granted bioRxiv a license to display the preprint in perpetuity. It is made available under aCC-BY 4.0 International license.

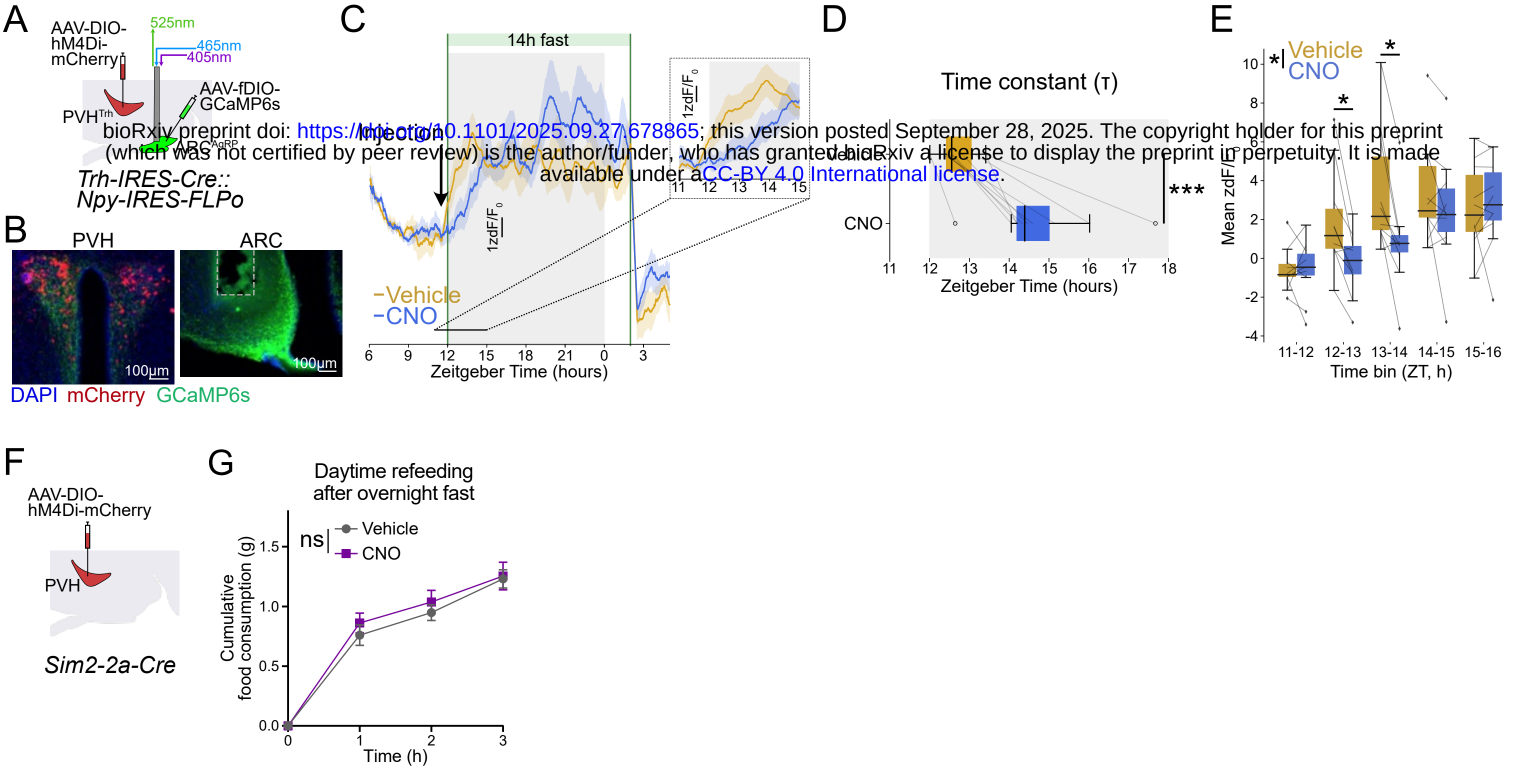

Figure S10

bioRxiv preprint doi: https://doi.org/10.1101/2025.09.27.678865; this version posted September 28, 2025. The copyright holder for this preprint (which was not certified by peer review) is the author/funder, who has granted bioRxiv a license to display the preprint in perpetuity. It is made available under aCC-BY 4.0 International license.

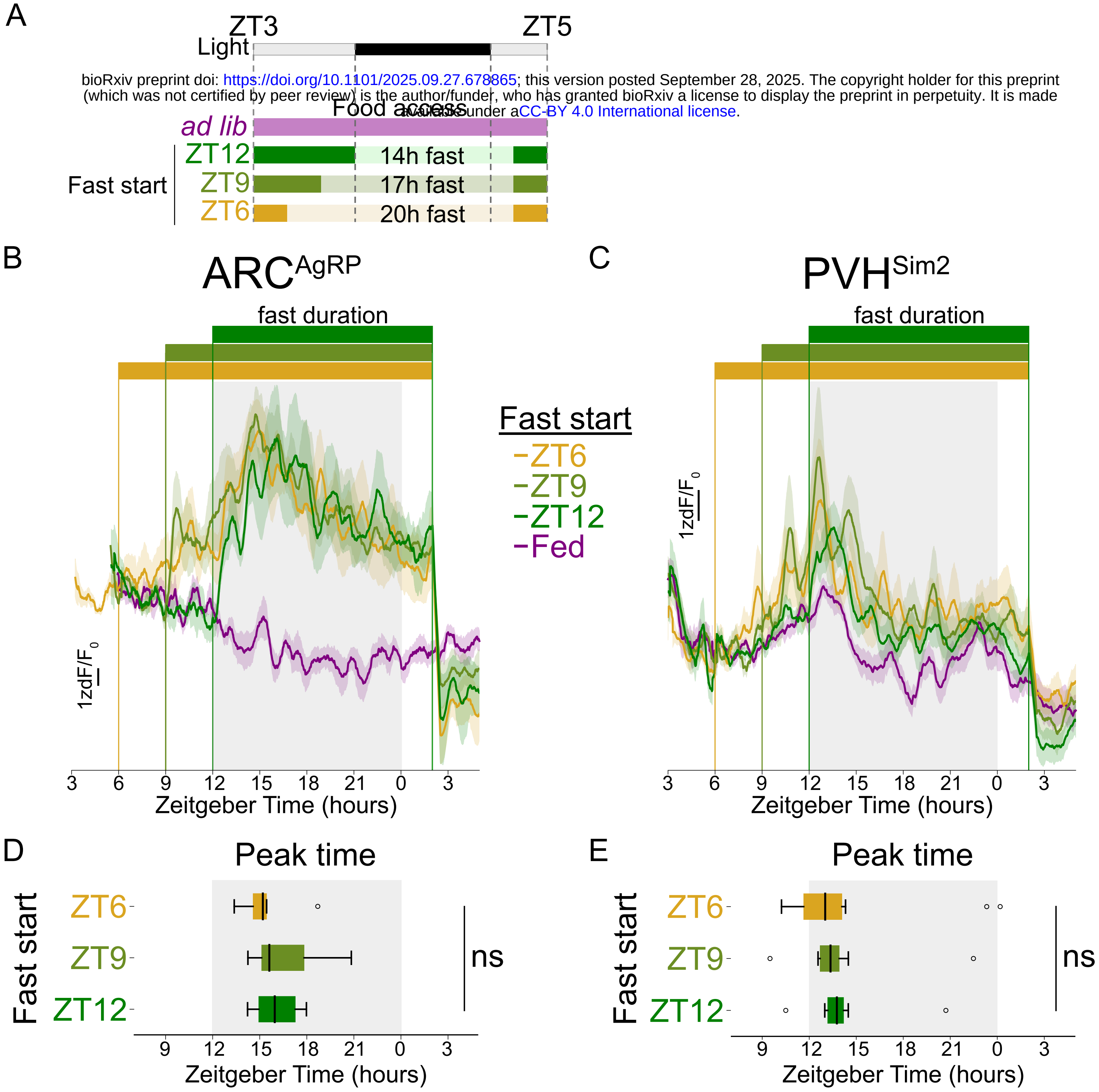

Figure S11

bioRxiv preprint doi: https://doi.org/10.1101/2025.09.27.678865; this version posted September 28, 2025. The copyright holder for this preprint (which was not certified by peer review) is the author/funder, who has granted bioRxiv a license to display the preprint in perpetuity. It is made available under aCC-BY 4.0 International license.

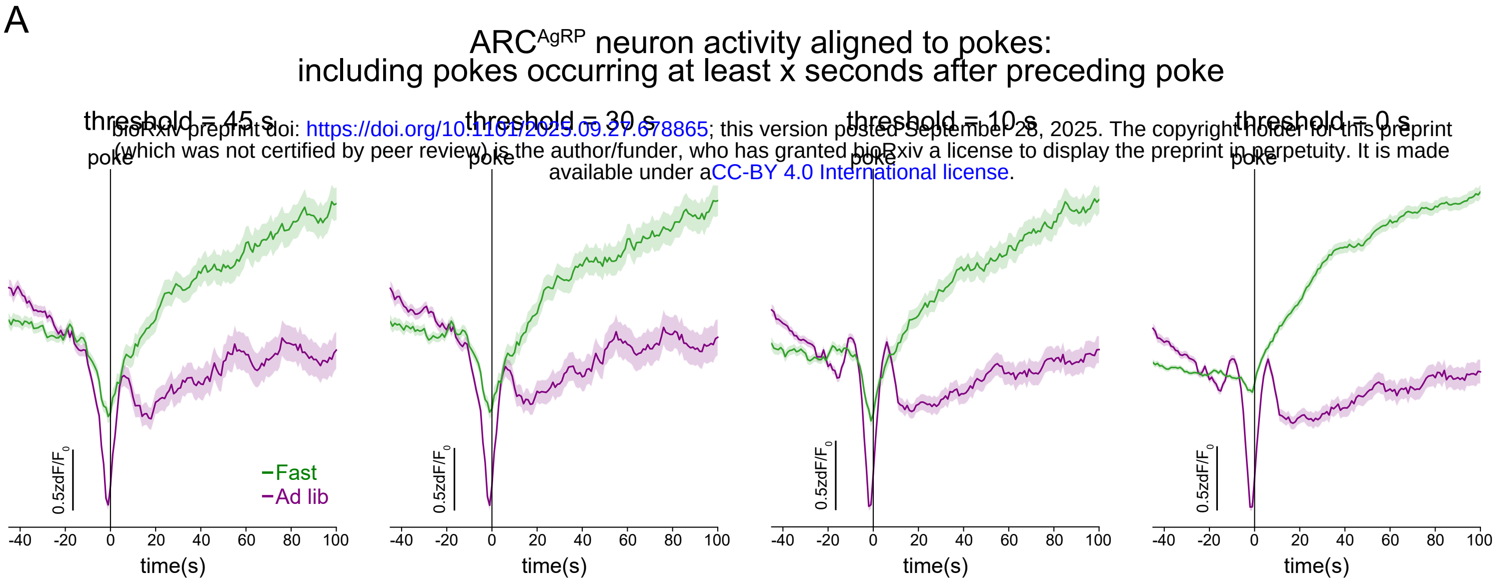

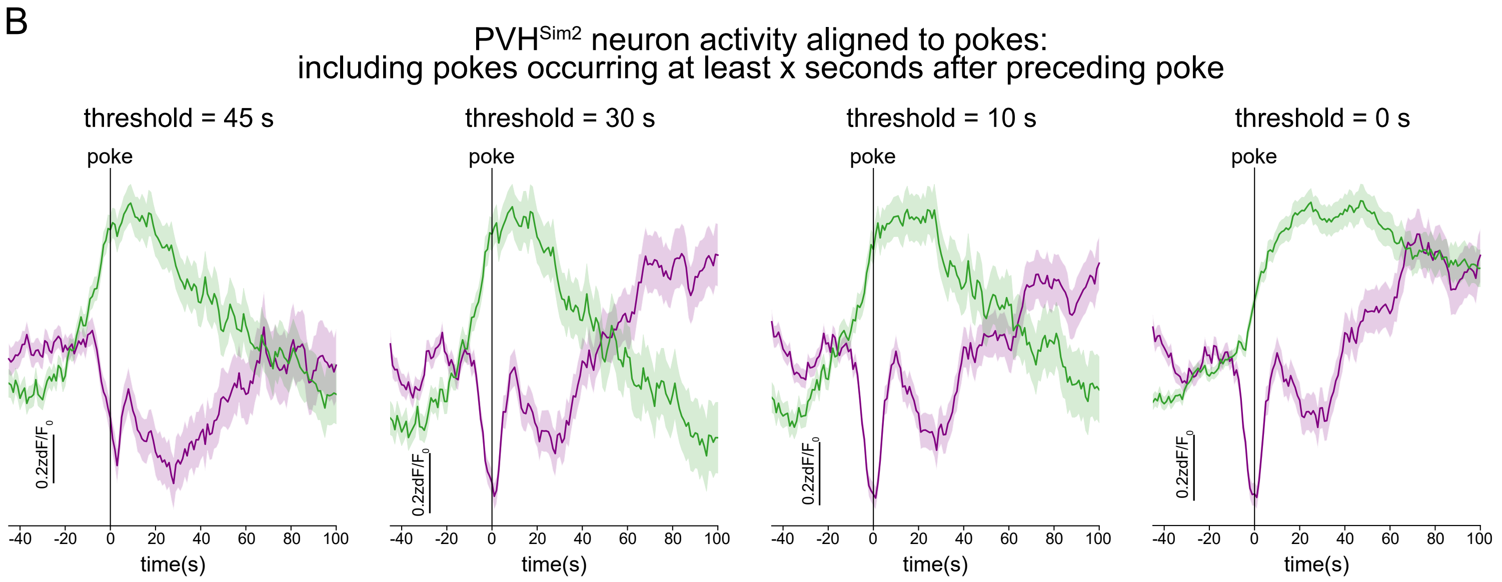

Figure S12

bioRxiv preprint doi: https://doi.org/10.1101/2025.09.27.678865; this version posted September 28, 2025. The copyright holder for this preprint (which was not certified by peer review) is the author/funder, who has granted bioRxiv a license to display the preprint in perpetuity. It is made available under aCC-BY 4.0 International license.

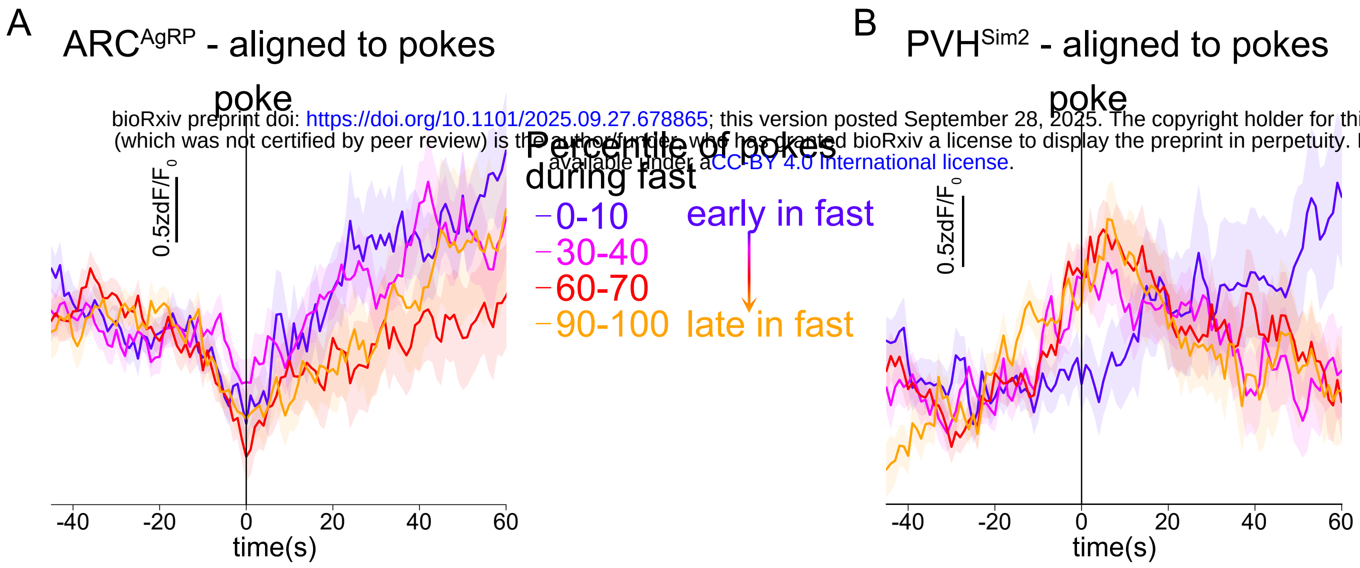

Figure S13

bioRxiv preprint doi: https://doi.org/10.1101/2025.09.27.678865; this version posted September 28, 2025. The copyright holder for this preprint (which was not certified by peer review) is the author/funder, who has granted bioRxiv a license to display the preprint in perpetuity. It is made available under aCC-BY 4.0 International license.

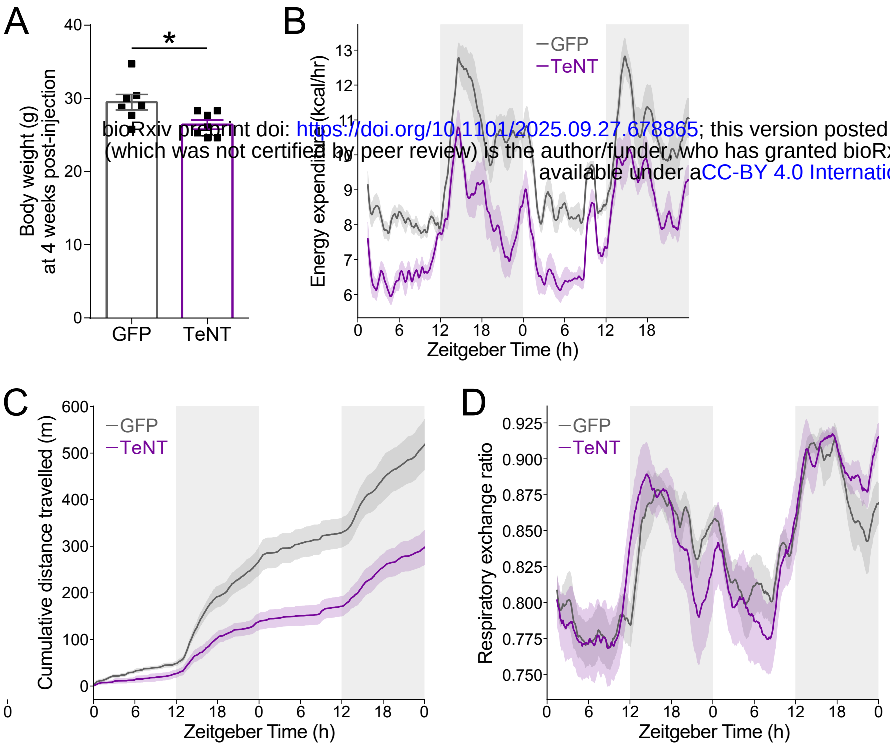

Figure S14

https://doi.org/10.1101/2025.09.27.678865 copyright holder for this preprint
preprint in perpetuity. It is made
CC-BY 4.0 International license

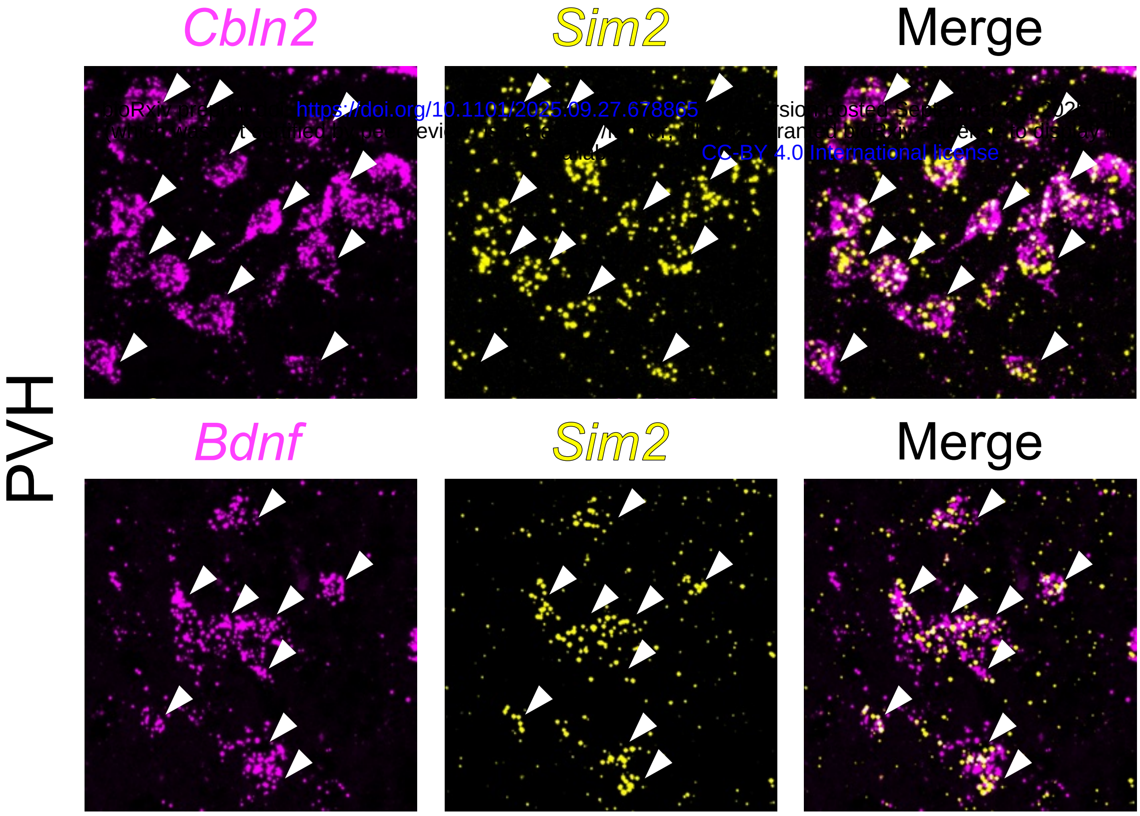

Figure S15
